# First- Versus Second-Generation Drug-Eluting Stents in Acute Coronary Syndromes (Katowice-Zabrze Registry)

**DOI:** 10.5935/abc.20160043

**Published:** 2016-05

**Authors:** Damian Kawecki, Beata Morawiec, Janusz Dola, Wojciech Waha, Grzegorz Smolka, Aleksandra Pluta, Kamil Marcinkiewicz, Andrzej Ochała, Ewa Nowalany-Kozielska, Wojciech Wojakowski

**Affiliations:** 1 2^nd^ Department of Cardiology - Zabrze Medical University of Silesia, Katowice -Poland; 2 3^rd^ Division of Cardiology - Katowice-Ochojec - Medical University of Silesia, Katowice - Poland

**Keywords:** Acute Coronary Syndrome, Drug-Eluting Stents, Thrombosis, Percutaneous Coronary Intervention

## Abstract

**Background:**

There are sparse data on the performance of different types of drug-eluting stents (DES) in acute and real-life setting.

**Objective:**

The aim of the study was to compare the safety and efficacy of first- versus second-generation DES in patients with acute coronary syndromes (ACS).

**Methods:**

This all-comer registry enrolled consecutive patients diagnosed with ACS and treated with percutaneous coronary intervention with the implantation of first- or second-generation DES in one-year follow-up. The primary efficacy endpoint was defined as major adverse cardiac and cerebrovascular event (MACCE), a composite of all-cause death, nonfatal myocardial infarction, target-vessel revascularization and stroke. The primary safety outcome was definite stent thrombosis (ST) at one year.

**Results:**

From the total of 1916 patients enrolled into the registry, 1328 patients were diagnosed with ACS. Of them, 426 were treated with first- and 902 with second-generation DES. There was no significant difference in the incidence of MACCE between two types of DES at one year. The rate of acute and subacute ST was higher in first- vs. second-generation DES (1.6% vs. 0.1%, p < 0.001, and 1.2% vs. 0.2%, p = 0.025, respectively), but there was no difference regarding late ST (0.7% vs. 0.2%, respectively, p = 0.18) and gastrointestinal bleeding (2.1% vs. 1.1%, p = 0.21). In Cox regression, first-generation DES was an independent predictor for cumulative ST (HR 3.29 [1.30-8.31], p = 0.01).

**Conclusions:**

In an all-comer registry of ACS, the one-year rate of MACCE was comparable in groups treated with first- and second-generation DES. The use of first-generation DES was associated with higher rates of acute and subacute ST and was an independent predictor of cumulative ST.

## Introduction

Drug-eluting stents (DES) were successfully introduced into clinical practice for percutaneous coronary interventions (PCI) as a response to high rate of restenosis associated with bare-metal stents (BMS).^1,2^ Pooled analyses from randomized studies with paclitaxel-eluting and sirolimus-eluting stents showed similar mortality and myocardial infarction (MI) rates, but less repeat revascularization in comparison to BMS.^3^ Older DES platforms, with relatively thick struts and durable polymers were however associated with late and very late stent thrombosis (ST).^4,5^

Current evidence shows that newer stent platforms with thinner struts, more biocompatible polymer and limus drugs provide better efficacy in terms of reduced thrombogenicity in preclinical studies as well as clinical safety (ST).^6,7^ Such stents are regarded as second-generation DES.

Both randomized trials and large registries have consistently shown improved safety and efficacy across patients subgroups, including acute coronary syndromes (ACS) and stable coronary artery disease (CAD).^8^ Stent thrombosis however, despite its slow rate, remains the main concern associated with the implantations of DES, especially in patients with high risk for bleeding, bad drug compliance and ACS due to the high mortality of this complication.^9^

The use of DES in ACS was initially off-label, however current guidelines indicate that DES should be preferred over BMS also in ACS including ST-segment elevation acute myocardial infarction (STEMI) based on randomized trials.^10-13^ The majority of studies that compared first- and second-generation DES patients with ACS consisted only a fraction of studied populations.^8,14-16^ In recent years a few studies comparing both generations of DES in acute setting were published.^17,18^ Nonetheless, these data are sparse and require further evaluation.

We therefore aimed to compare the safety and efficacy of first-generation vs. second-generation DES in all-comer ACS population in one-year follow-up.

## Methods

### Study design

The investigator-initiated all-comer Katowice-Zabrze Registry involved consecutive patients treated with PCI with implantation of DES. The enrollment was conducted in two tertiary high volume (together 5500 PCI/year) cardiac centers (Upper Silesian Medical Center in Katowice and 2nd Department of Cardiology, Zabrze) from January, 1^st^ 2009 to December, 31^st^ 2010. The aim of this ongoing registry is to compare the first and second generations of DES in unrestricted population of patients. Within the registry population, the inclusion criterion was the diagnosis of ACS treated with PCI with the implantation of either first- or second-generation DES. ACS was defined according to the current guidelines as unstable angina (UA), non-ST-elevation MI (NSTEMI) or STEMI.^19-21^ In coronary angiography, the basic angiographic characteristics were recorded: location of the lesion, severity of stenosis, AHA/ACC lesion type, thrombus, calcifications. In every patient, excluding those after coronary artery bypass grafting (CABG), the SYNTAX score was assessed. Stents were chosen out of first-generation DES durable polymer based or second-generation DES, according to the operator's decision. In case of the implantation of more than one stent in one patient, the DES implanted to culprit lesion or to more severe stenosis was considered as the index procedure. Dual antiplatelet therapy (acetylsalicylic acid and P2Y_12_ subtype of ADP receptor inhibitors) was prescribed for up to 12 months after the procedure in each patient. Baseline clinical, angiographic and procedure related data were retrospectively collected from medical records.

### Coronary stenting

Stents for implantation were chosen from first-generation DES durable polymer based [Paclitaxel-eluting stents (PES) (Taxus, Boston Scientific Corporation, Maple Grove, MN, USA) or Sirolimus-eluting stent (SES) (Cypher, Cordis, USA)] or second-generation DES [Everolimus-eluting stent (EES) (Promus, Boston Scientific Corporation; Xience, Xience Prime, Abbott Vascular, Santa Clara, CA, USA), Zotarolimus-eluting stent (ZES) (Endeavor, Resolute, Medtronic, Minneapolis, MN, USA), and Biolimus-eluting stent (BES) (Biolimus A9, Biosensors International, Switzerland)].

### Antiplatelet and antithrombotic regimen

All patients were treated according to guidelines for ACS and received a loading dose of aspirin and ADP-receptor inhibitor prior, during or directly after PCI, and a bolus of unfractionated heparin prior to PCI. IIb/IIIa receptor inhibitor was administered according to operator's decision. Following the procedure, patients were prescribed aspirin, 75 mg daily, lifelong, and clopidogrel, 75 mg daily, for up to 12 months, which was modified in patients who required anticoagulation therapy for other reasons.

### Follow-up

Patients were followed up at one year. All information was obtained from medical records of enrolling centers. If no information was available, phone contact was attempted. In case of phone contact failure, information on clinical endpoints was obtained from National Health Care System.

The primary efficacy endpoint was a composite major adverse cardiac and cerebrovascular events (MACCE) including all-cause death, non-fatal MI, target-vessel revascularization (TVR), and stroke.

The secondary endpoints were individual components of the primary endpoint: all-cause death, MI, TVR, stroke, as well as CABG. The safety of DES was defined as definite ST (acute, subacute, late and cumulative) and gastrointestinal bleeding rate at one year. MI was defined according to the universal definition.^19^ TVR, definite ST, acute, subacute and late ST were defined according to the definitions of endpoints for clinical trials.^22^ Gastrointestinal bleeding was considered an endpoint if fulfilled criteria of type 3 or type 5 bleeding, according to proposed definitions.^23^

Informed consent was obtained from each patient and the study protocol conforms to the ethical guidelines of the 1975 Declaration of Helsinki as reflected in a priori approval by the Ethical Committee of the Medical University of Silesia (No. KNW/0022/KB/59/11).

### Statistics

Variables were checked for normality of distribution with Shapiro-Wilks test. Continuous variables are presented as mean ± SD or median (25^th^, 75^th^ percentile) and were compared with Student *t* test or Mann-Whitney test. Categorical variables are presented as percentages and were compared with chi-square test. The Kaplan-Meier survival curves were constructed to describe the incidence of endpoints over time. The assessment of influence of parameters significantly statistically different between groups on endpoints was conducted with univariate Cox analysis. Multivariate Cox regression model was used to identify risk factor for safety and efficacy endpoints and included all variables statistically significant in univariate analysis. All tests were two-tailed and the value of p < 0.05 was considered significant. Analysis was performed with Statistica software, version 10PL (StatSoft Inc., Tulsa, OK, USA) and GraphPad Prism, version 6.00 (GraphPad, La Jolla, California, USA).

## Results

A total of 8284 PCI were performed during analyzed period. Of them, 6368 patients who received BMS (6177 patients) or underwent balloon angioplasty (191 patients) were excluded. Out of remaining 1916 patients who underwent PCI with the implantation of DES, 588 patients had stable CAD and were excluded from the analysis. Remaining 1328 patients were diagnosed with ACS (including 131 STEMI, 285 NSTEMI, and 912 UA patients) and subjected to the current analysis. Of them, 426 were treated with first-generation DES (391 PES, 35 SES) and 902 with second-generation DES (90 BES, 483 EES, 329 ZES). The distribution of initial diagnosis in both groups is presented in Table 1.

**Table 1:** Clinical characteristics.

**Characteristic**	**First-generation DES (n = 426)**	**Second-generation DES (n = 902)**	**P value**
Male sex	255 (60)	590 (65)	0.05
Age (years)	64 ± 9.4	63.2 ± 10.4	0.17
BMI (kg/m^2^)	29.3 ± 4.9	28.8 ± 4.7	0.26
Obesity	103 (24)	198 (22)	0.37
Renal insufficiency	75 (18)	171 (19)	0.56
Ejection fraction (%)	50 (42;55)	54 (45;60)	0.03
Diabetes mellitus	171 (40)	331 (37)	0.23
Hypertension	360 (85)	790 (88)	0.12
Dyslipidemia	278 (65)	575 (64)	0.59
Smoker	99 (23)	212 (24)	0.92
Familial history of CAD	133 (31)	317 (35)	0.16
Prior AMI	179 (42)	439 (49)	0.02
Prior PCI	220 (52)	478 (53)	0.65
Prior CABG	90 (21)	225 (25)	0.13
Carotid atherosclerosis	19 (4)	57 (6)	0.17
PAD	51 (12)	105 (12)	0.86
**Initial diagnosis**			
Unstable Angina	265 (62)	647 (71)	< 0.001
NSTEMI	109 (26)	176 (20)	0.01
STEMI	52 (12)	79 (9)	0.05

Data are presented as n (%), median (25^th^ and 75^th^ interquartile) or mean±SD. DES: drug eluting stent; BMI: body mass index; CAD: coronary artery disease; AMI:acute myocardial infarction; PCI: percutaneous coronary intervention; CABG: coronary artery bypass grafting; PAD: peripheral artery disease; NSTEMI: non-ST-segmentelevation myocardial infarction; STEMI: ST-segment elevation myocardial infarction.

Both groups had similar baseline profile (Table 1). Comparable rates of cardiovascular risk factors were observed. Patients who received a second-generation DES had higher incidence of prior acute MI than patients with first-generation DES (49% vs. 42%, p = 0.02). Patients' history of coronary interventions did not differ significantly between groups.

**Figure 1:**
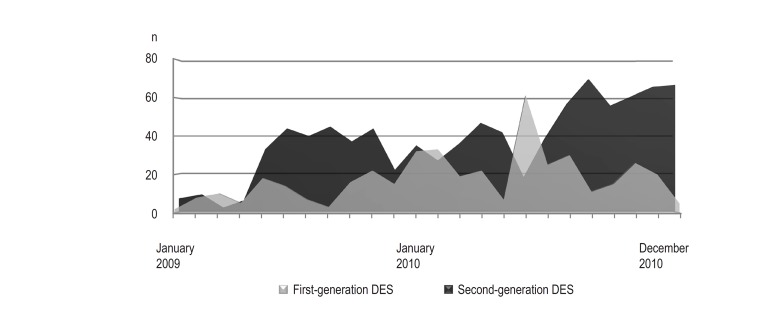
Temporal distribution of the number of first- and second-generation DES implanted during studied period. DES: drug-eluting stent.

Angiographic and procedural characteristics are depicted in Table 2. No differences regarding treated vessel were found between groups. Higher SYNTAX score was observed in first-generation than in second-generation DES (median 17 vs. 13 points, p < 0.001). Thrombus and calcifications were more commonly found in first-generation DES (p < 0.001 and p < 0.001, respectively). Temporal distribution of the implantation of both types of DES during studied period is presented in Figure 1. First-generation DES were implanted more frequently after predilation and with lower mean inflation pressure than second-generation DES (p = 0.002 and p < 0.001, respectively). Procedures did not differ regarding length and diameter of the stent, as well as total number of stents per lesion. Angiographic outcome of the procedure was equal, and TIMI 3 flow was achieved in 98% of cases in both groups (p = 0.48). Regarding antithrombotic and antiplatelet treatment, IIb/IIIa receptor inhibitors were administered in 7% and 6% of cases in first- and second-generation DES, respectively (p = 0.62). Aspirin was prescribed in 99% and 98% of patients in first- and second-generation DES group, respectively (p = 0.23). Patients received oral anticoagulation with equal frequency (6%) in both groups (p = 0.8). Among them, in 3 patients from first-generation DES group (0.7%) and in 1 patient from second-generation DES group (0.1%), aspirin was discontinued after 3-6 months (p = 0.19).

**Table 2:** Angiographic and procedural characteristics.

**Characteristic**	**First-generation DES (n = 426)**	**Second-generation DES (n = 902)**	**P value**
**Culprit vessel**			
LM	40 (9)	59 (7)	0.07
LAD	216 (51)	442 (49)	0.56
Cx	69 (16)	158 (18)	0.55
RCA	78 (18)	186 (21)	0.32
SVG	21 (5)	49 (5)	0.7
AG	2 (0.5)	8 (1)	0.41
SYNTAX score	17 (10;28)	13 (7;22)	< 0.001
Thrombus	30 (7)	26 (3)	0.001
Ostial lesion	74 (18)	128 (15)	0.25
Restenosis	72 (17)	144 (16)	0.67
Calcifications	56 (13)	36 (4)	< 0.001
Stenosis severity (%)	86.8	87.4	0.78
No DES per lesion	1 (1;1)	1 (1;1)	0.15
Length DES per lesion (mm)	22 (15;29)	22.5 (15;28)	0.57
Stent diameter (mm)	3.03 ± 0.48	3.07 ± 0.47	0.55
Predilation	222 (53)	368 (44)	0.002
Maximal inflation pressure (atm)	16 ± 4	17 ± 4	< 0.001
TIMI 3 flow	419 (98)	881 (98)	0.48
GPIIb/IIIa inhibitors	28 (7)	53 (6)	0.62

Data are presented as n (%), median (25^th^ and 75^th^ interquartile) or mean±SD. DES: drug eluting stent; LM: left main; LAD: left anterior descending artery; Cx:circumflexartery; RCA: right coronary artery; SVG: saphenous graft; AG: arterial graft; TIMI: thrombosis in myocardial infarction.

### Endpoints

**Figure 2:**
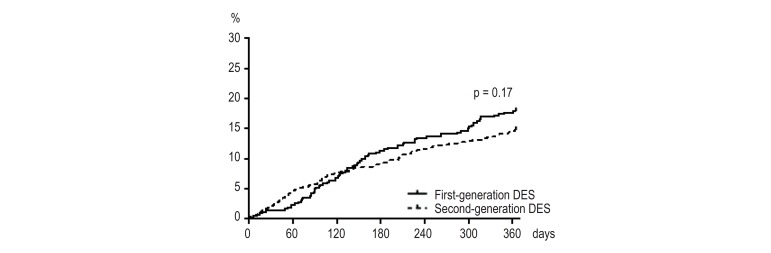
Incidence of MACCE at 1 year. MACCE: major adverse cardiac and cerebrovascular events; DES: drug-eluting stents.

There was no significant difference in the incidence of the primary and secondary efficacy endpoints between first- and second-generation DES at one year (Table 3). The Kaplan-Meier curves for the incidence of MACCE are presented in Figure 2 with no significant difference between groups. In univariate Cox regression model, the predictors of the incidence of MACCE were left ventricular ejection fraction, history of acute MI, SYNTAX score and predilation (Table 4). After adjustment in multivariate analysis only the history of acute MI was a statistically significant predictor of MACCE (HR 1.39, CI 1.04-1.84, p=0.03) (Table 5).

**Table 3:** Clinical outcomes at one year.

**Characteristic**	**First-generation DES (n = 426)**	**Second-generation DES (n = 902)**	**P value**
**Stent thrombosis (ST)**			
Acute ST	7 (1.6)	1 (0.1)	< 0.001
Subacute ST	5 (1.2)	2 (0.2)	0.025
Late ST	3 (0.7)	2 (0.2)	0.18
Cumulative ST	15 (3.5)	5 (0.6)	< 0.001
**Primary endpoint**			
MACCE	80 (19)	135 (15)	0.078
**Secondary endpoint**			
Death	19 (4.5)	39 (4.3)	0.91
AMI	31 (7.2)	43 (4.8)	0.06
TVR	51 (12)	90 (10)	0.27
Stroke	6 (1.4)	5 (0.6)	0.11
CABG	12 (2.8)	12 (1.3)	0.06
Gastrointestinal bleeding	9 (2.1)	10 (1.1)	0.15

Data are presented as n (%). DES: drug eluting stent; ST: stent thrombosis; MACCE: major adverse cardiac and cerebrovascular events; AMI: acute myocardialinfarction; TVR: target vessel revascularization; CABG: coronary artery bypass grafting.

**Table 4:** Univariate Cox proportional hazard model for the incidence of MACCE and ST.

**Characteristic**	**p value**	**HR**	**HR CI**		**p value**	**HR**	**HR CI**
	**MACCE**				**Cumulative stent thrombosis**		
First-generation DES	0.07	1.29	0.98-1.7		< 0.001	4.61	1.88-11.31
Sex (male)	0.38	1.13	0.86-1.5		0.36	1.53	0.6-3.91
Prior AMI	0.005	1.46	1.12-1.94		0.34	0.66	0.27-1.56
LVEF	0.04	0.99	0.98-0.999		0.04	0.97	0.94-0.998
SYNTAX Score	0.02	1.02	1.0-1.03		< 0.001	1.06	1.03-1.09
Thrombus	0.99	1.0	0.53-1.89		< 0.001	6.99	2.57-18.97
Calcifications	0.79	1.08	0.61-1.94		0.22	2.13	0.63-7.24
Predilation	0.009	1.44	1.09-1.91		0.03	2.83	1.1-7.28
Max inflation pressure	0.52	0.98	0.94-1.03		0.02	0.83	0.72-0.97

DES: drug eluting stent; AMI: acute myocardial infarction; LVEF: left ventricular ejection fraction; CI: confidence interval.

**Table 5:** Multivariate Cox proportional hazard model for the incidence of MACCE.

**Characteristic**	**p value**	**HR**	**HR CI**
**MACCE**			
Prior AMI	0.03	1.38	1.04-1.84
LVEF	0.65	0.98	0.98-1.01
SYNTAX Score	0.38	1.01	0.99-1.02
Predilatation	0.05	1.34	1.0-1.79

DES: drug eluting stent; AMI: acute myocardial infarction; LVEF: left ventricular ejection fraction; CI: confidence interval.

**Figure 3:**
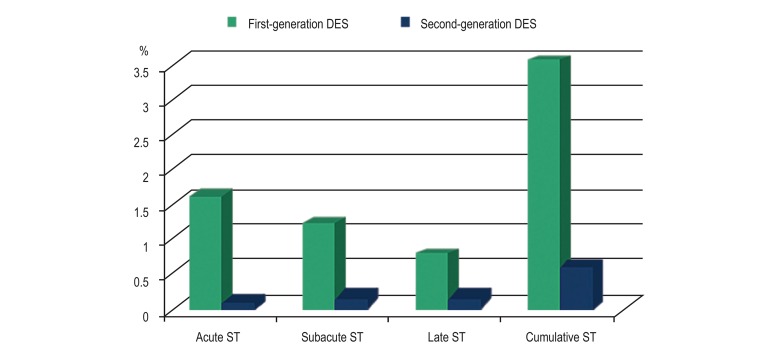
Stent thrombosis (ST) rates. ST: stent thrombosis; DES: drug-eluting stents.

Regarding the safety profile, the rate of acute and subacute ST was significantly higher in first- than in second-generation DES (1.6% vs. 0.1%, p < 0.001 and 1.2% vs. 0.2%, p = 0.025, respectively) (Figure 3). There was no significant difference between first- and second-generation DES in the occurrence of late ST (0.7% vs. 0.2%, respectively, p = 0.18) and gastrointestinal bleeding (2.1% vs 1.1%, respectively, p = 0.21). Cox regression model for the incidence of cumulative ST revealed that, among other parameters, the first generation of DES was an independent predictor in univariate analysis (HR 4.61, CI 1.88-11.31, p < 0.001) (Table 4).

## Discussion

The Katowice-Zabrze registry shows that, in patients with ACS treated with PCI, the use of second-generation DES might be associated with better safety profile, and lower rate of acute and subacute ST at one year. There was, however, no difference in favor of second-generation DES as to the overall MACCE rate.

Similar observations for the population of ACS have been published previously,^18,24^ suggesting that, for the treatment of STEMI, all (first- and second-generation) DES show similar results, notwithstanding higher late lumen loss, restenosis and thrombosis rates for first-generation DES. It seems that the PCI in ACS is similarly efficient regardless of the type of eluting drug.

Of note, the rates of MACCE in our population were higher than those presented earlier by different groups.^17,25^ This could be explained by the differences in the profile of the population with more or less restricted criteria of enrollment (exclusion of the implantation of DES due to ST or patients in cardiogenic shock, with renal insufficiency or with suboptimal outcome of the index procedure). Lower overall endpoint rate for patients with ACS and lower incidence of MACCE for first-generation DES than in our study was also reported in a pooled analysis of 4 randomized trials.^7^ The reason for this could be different profile of the population with higher rates of risk factors (diabetes mellitus, arterial hypertension, prior acute MI and prior PCI), more complex lesions (more left anterior descending and left main coronary arteries as the indexed procedure, longer lesion, higher diameter of stenosis) than in our cohort.

Finally, high rates of endpoints in our study could be explained in a comparison of the trial with the most consistent inclusion criteria with ours, i.e. the SORT OUT-III trial.^16^ The SORT OUT was a randomized trial with a great fraction of non-randomized patients, thus not undergoing the analysis. Better risk profile than presented here had implication in lower rates of endpoints in the studied population. Our study is an analysis of an all-comer, unrestricted and independent use of DES in real-life ACS population, thus its outcomes could reflect real clinical practice and could be directly applied into patient care.

All-comer Swedish SCAAR Registry with more than 94000 patients showed that second-generation DES have 62% less risk of ST than BMS and 43% less than first-generation DES, which is consistent with our data. In large SCAAR population there was also reduction of mortality in favor of second-generation DES.^8^ The observations were confirmed by network meta-analysis by Palmerini et al., showing in pooled analysis of 49 randomized clinical trials with 50,844 patients a consistent reduction of ST with new generation DES in comparison to first-generation DES and BMS.^26^

Regarding the safety of DES, ST is the most serious and often fatal form of target-vessel failure. Nevertheless, the percentage of TVR of thrombotic origin reported here is low. Presented results confirm significantly higher occurrence of the thromboembolic complication in short-term follow-up after implantation of first-generation DES. These facts are not surprising, considering the majority of previously published data.^8,27,28^ Higher rates of acute and subacute ST were observed despite no difference in post-procedural angiographic characteristics, and no difference in the administration of standard in-hospital dual antiplatelet therapy. Higher rates of ST in first- than second-generation DES could be explained by significantly higher CAD burden in this group as measured by the SYNTAX score, although classifying patients in both groups as low risk with median score < 22.

It is an interesting observation that only rates of acute and subacute ST were significantly different between first- and second-generation DES and the cumulative ST rate was driven by early ST events. Several differences in stent design might be attributable for these differences, namely impaired strut endothelialization in first-generation stents related to higher strut thickness, less biocompatible polymer coating (polyolefin derivative in Taxus and PEVA + PBMA copolymer in Cypher) causing peri-strut inflammatory response, polymer structural defect after deployment as well as paclitaxel which may cause delayed endothelial recovery. New generation EES were shown to be less susceptible to inflammatory response and thrombosis.^29^ Of course the optimization of the procedure with proper stent sizing and deployment is equally important, especially in patients with ACS and high thrombotic burden.^30^

These differences were not reflected in the clinical follow-up, with similar rates of MACCE in both groups. According to the publications in this field,^31,32^ the major concern accompanying the implantation of DES is very late ST. Lack of the routine angiographic follow-up and the observational period restricted to one year in the present study limit the possibilities for deeper understanding of clinical significance of the two major in-stent complications, ST and restenosis, and their interaction over time. It is known that ST in BMS occurs entirely due to restenosis.^33^ The thrombotic origin of TVR in DES is a derivative of several factors,^34^ such as the characteristics of the lesion specific for ACS.

### Limitations

The study is retrospective and observational in nature, thus saddled with obvious limitations. Lack of random allocation to receive either first- or second-generation DES resulted in disproportion of the type of ACS in each group and, despite equal STEMI rates regarded as the strongest factor for ST, might have affected the results. The safety endpoint was defined as definite ST. This could underestimate real incidence of ST in follow-up. However, according to Cutlip et al.,^22^ the quality of data, which were received from the follow-up of this retrospective registry, had to be taken into account. In case of acute MI occurrence in the follow-up, there was no possibility of checking if there was documented acute ischemia in the territory of the implanted stent. In cases where coronary angiography was accessible, it was verified and classified as definite ST if applicable. Finally, one of the most prone conditions to the development of ST is incomplete strut apposition. No routine use of an intracoronary imaging technique after stent placement, reflecting retrospective nature of the study, does not render precise indication of operator- or stent-related cause of stent failure.

## Conclusions

In this all-comer registry of ACS patients, the 12-month MACCE rate was comparable in groups treated with first- and second-generation DES. The use of first-generation DES, as an independent predictor of cumulative ST, was associated with higher rates of acute and subacute ST, but similar rate of late ST and gastrointestinal bleeding when compared with the use of second-generation DES.
