# Erratum

**DOI:** 10.5935/abc.20160121

**Published:** 2016-08

## Issue of April 2016, vol. 106 (4), Supl. 2, pp. 1-22

In "II Diretrizes Brasileiras de Fibrilação Atrial", consider Kuniyoshi RR as the correct form for the name of the author Ricardo Ryoshim Kuniyoshi.

## Issue of May de 2016, vol. 106 (5), pp. 373-381

In the original article "First- Versus Second-Generation Drug-Eluting Stents in Acute Coronary Syndromes (Katowice-Zabrze Registry)", adjust the name of author Wojciech Waha to Wojciech Wanha.

